# Pore Size Distribution in Granular Material Microstructure

**DOI:** 10.3390/ma10111237

**Published:** 2017-10-27

**Authors:** M. Mahdi Roozbahani, Rodrigo Borela, J. David Frost

**Affiliations:** 1 Computational Science and Engineering, Georgia Institute of Technology, Atlanta, GA 30332, USA; mahdir@gatech.edu (M.M.R.); rborelav@gatech.edu (R.B.); 2 Civil and Environmental Engineering, Georgia Institute of Technology, Atlanta, GA 30332, USA

**Keywords:** pore size distribution, 3D segmentation algorithm, digital material representation, granular material characterization

## Abstract

Pore scale modeling plays a key role in fluid flow through porous media and associated macroscale constitutive relationships. The polyhedral shape and effective local pore size within granular material microstructure are computed in this study by means of the Euclidean Distance Transform (EDT), a local maxima search (non-maximum suppression), and a segmentation process. Various synthetic packed particles are simulated and employed as comparative models during the computation of pore size distribution (PSD). Reconstructed un-sheared and sheared Ottawa 20–30 sand samples are used to compute PSD for non-trivial and non-spherical models.

## 1. Introduction

Pore space modeling is a challenging issue for scientists in various disciplines. Granular structures yield complex pore geometries, not easily accessible experimentally and difficult to characterize using non-idealized computational methods. Nonetheless, insight into the pore space is essential to homogenization techniques and the macroscale constitutive modeling of flow through porous media [1,2,3]. Fluid flow takes place within inter-connected pores, which govern seepage, drainage, consolidation, and internal stability [4,5]. In granular filters, which have their drainage capability severely impacted by clogging, it is fundamental to define the pore space precisely [6,7,8]. Currently, a variety of approaches are available for computing the pore size distribution (PSD) mainly categorized in Delaunay tessellation, medial axis, and watershed based methods [9,10,11,12,13,14,15,16,17,18].

In the Delaunay tessellation method [19], a pore is delimited by a tetrahedron with vertices at the centers of four nearest neighboring particles. The effective pore size is then defined as the diameter of the largest inscribed sphere in this tetrahedron. This formulation encompasses two major limitations: (1) reasonable results are only obtainable for packings of spherical particles; and (2) pores bounded by more than four spheres cannot be captured, resulting in a dense tessellation network that often leads to multiple subdivisions of a single pore. In the medial axis approach [9], the skeleton of a continuum pore space is first identified. Subsequently, individual pores are located through a series of merging steps of spherical voids centered along the medial axis. Despite not being restricted by granular arrangement or particle shape, the method suffers from a similar limitation to Delaunay tessellation, in that voids of complex polyhedral shapes cannot be accurately reproduced by the algorithm. In watershed methods [17], the pore space is first converted to an Euclidean distance field [20]. Voxels are then sequentially ascribed to a catchment basin starting at the local maxima until different expansion fronts collide and form the boundaries that constitute the segmentation. While capable of capturing a range of pore geometries, the method often results in over-segmentation [21], requiring arbitrarily set parameters to achieve successful results [22].

In this study, a new adaptive method is proposed to discretize the pore space and compute the PSD consistent with a diverse range of void shapes and unrestrained by the need of user-defined parameters. The approach consists of three steps. First, similarly to watershed methods, a binarized solid-void digital image is converted into a new domain by applying the Euclidean distance transform (EDT) [20]. Subsequently, pore centers are identified utilizing a robust non-maximum suppression routine [23]. Lastly, a segmentation process (minimum Euclidean distance assignment) [24] is applied to discriminate individual pores, irrespective of shape or size, in the originally continuous void space. Additionally, pore shape and orientation is explored, using the Principal Component Analysis (PCA) technique proposed by Wijewickrema and Paplinski [25].

For verification purposes, spherical packings of known PSD were analyzed employing the method introduced herein. Following a successful validation, the tool was then applied to numerically generated packings at different density states. Random loose packings were generated by inserting spheres one at a time and allowing them to roll until a stable arrangement was reached [26,27,28]. Denser packings were obtained following a newly formulated procedure for cylindrical domains (Section 2.1), in which randomly placed overlapping spheres are subjected to a series of micro-rearrangements that yield a denser state [29,30,31].

Finally, the soundness of the PSD characterization technique described in this paper is demonstrated through its application to 3D digital models of Ottawa 20–30 sand specimens [32,33].

## 2. Synthetic Specimen Generation

The macro behavior of granular materials is highly dependent on particle arrangement, local and overall porosity [34,35,36]. While extensive research has shown that granular materials can be reproduced numerically [19,29,37,38], careful attention should be paid to the packing generation method to achieve reasonable equivalence between physical and simulated materials. Accordingly, a thorough discussion of the methods employed in the present study follows. In general, the structure is represented as random packings of spherical particles, produced via dynamic or geometrical methods. In dynamic packing simulations, particles are subject to Newton’s second law of motion and contacts are treated as a linear-spring-dashpot system, after the method developed by Cundall [39], known as the Discrete Element Method (DEM). While DEM can produce any desired randomly packed structure in a predefined density range, particles are allowed to overlap, which requires a high contact stiffness to be assigned in order to avoid numerical artifacts. Since the time step is inversely proportional to contact stiffness, the number of iterations necessary to reach convergence becomes substantially greater. Given DEM’s inextricable computational expense [40], such added cost can render simulations intractable. The computational advantage of the presented approach has been discussed in a related study [16]. Similar benefits have been demonstrated by others [41,42].

This study focuses on the characterization of geomaterials, which are generally well-sorted and uniform in size. Therefore, for the comparative study of the pore size distribution presented herein, it was deemed suitable to analyze four mono-sized sphere packings, each pertaining to a different class following Dullien’s classification [43] shown in Table 1. In this paper, the terms density and solid ratio are used interchangeably.

### 2.1. Generation Method

Packings classified as ”very loose random” according to Table 1 were obtained via the algorithm developed in the previous study by Roozbahani et al. [27]. This method is initiated by randomly generating initial (x,y,z) coordinates for every sphere according to a specific distribution. This determines the initial behavior of every sphere (e.g., collision, rotation, or relative stability with the other spheres).

In order to generate denser specimens, an algorithm was developed to closely pack hard spheres through a series of steps, which can be divided into four main stages: initialization of sphere coordinates, particle expansion/contraction, overlap reduction, and vibration.

Commonly, initialization is done by assigning spheres random positions within the domain, often encompassing an excessive number of anomalously large overlaps. A suitable alternative is to utilize a random loose packing [27] as the initial input for spheres coordinates. By starting the algorithm with an overlap-free packing, several unnecessary overlap reduction and vibration steps are eliminated, improving the overall efficiency.

Post-initialization, the packing is densified by imposing a small (2.5% of $radius_{particle}$) uniform particle expansion. The overlap reduction stage ensues, according to the procedure proposed by He et al. [29]. In each iteration, particles are moved in the direction of an overlap-minimizing vector, and the maximum global overlap (MGO) is calculated. This process is comparable to the particle rearragements produced in DEM simulations by repulsion forces at particle contacts. Nonetheless, the efficiency is much higher since geometrically updating particle positions dispenses the use of a Newtonian time integration scheme [44], necessary in dynamic simulations. Cycling of the overlap-reduction phase is interrupted under two circumstances: the MGO decreases below the intermediate tolerance $T_{i}$, or the number of iterations surpasses the established limit $i_{\max}$. In the first case, spheres are enlarged, and the system is once again subjected to a stage of overlap reduction. Conversely, when the cycling limit is exceeded ($iteration>i_{\max}$), a slight contraction is applied to all particles. The packing density is then determined within a virtual container. If the desired density state has been reached, the simulation is stopped and sphere coordinates are recorded. Otherwise, the global overlap tolerance $T_{i}$, initially set at 0.01, is decreased to accelerate the process, and the specimen is submitted to the overlap reduction process again.

The density is computed inside a virtual rectangular container [19], so as to exclude the zone influenced by boundary effects. This zone was shown to dissipate at a distance no larger than $4\;radius_{particle}$ from the walls [16].

**Figure 1:**
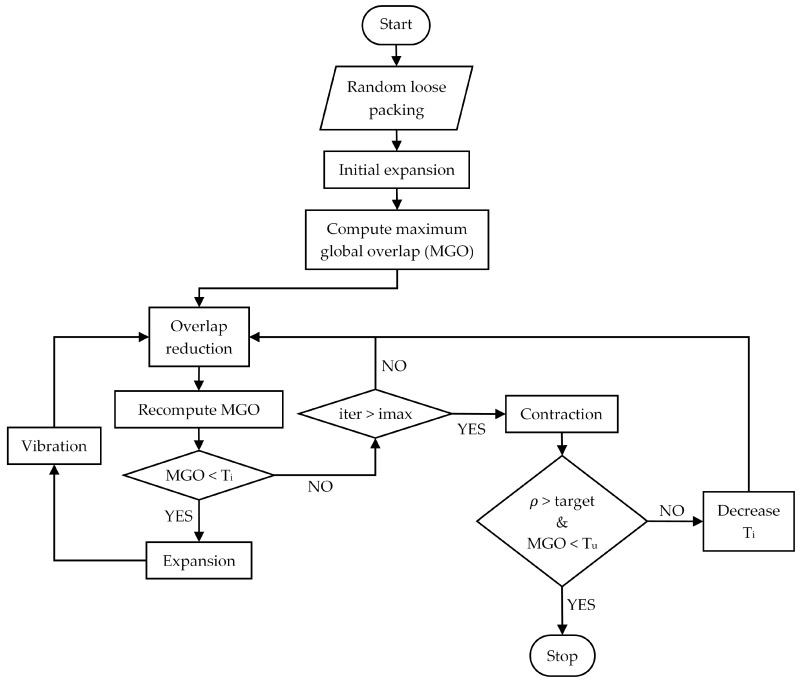
Flowchart of random close packing algorithm.

Due to the quasi-deterministic nature of the overlap reduction process, particles can become jammed, thereby hindering convergence. Hence, a vibration stage was introduced post-particle expansion. This step, which was originally implemented after Clarke and Wiley [31], consists of moving particles in the direction of a random vector. The resulting perturbations promote local rearrangement that releases trapped particles and advances the packing towards its target state. The combination of these steps, allowed tangible improvement of the computational cost. A complete flowchart of the algorithm is shown in Figure 1.

**Figure 2:**
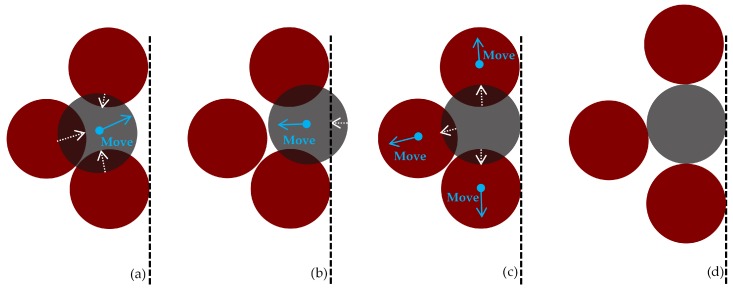
Schematic representation of sphere-wall interaction. (**a**) selected sphere movement based on overlap-minimizing vector; (**b**) selected sphere being moved in direction of central axis; (**c**) moving overlapping spheres to tangent position with selected sphere; (**d**) final arrangement.

Most available geometric packing methods rely on periodic boundary conditions to avoid handling particle-wall interactions, thus restricting simulations to only producing cuboid specimens [45,46,47,48]. The present study addresses this impediment by developing a particle-wall interaction scheme (Figure 2), which enables specimens to be generated within cylindrical containers with ($\frac{radius_{container}}{radius_{particle}}\geq 10$). Figure 2a illustrates the overlap reduction stage, in which a selected sphere is moved and made tangent to its intersecting neighbors. As the new position may lead to an overlap with the container’s boundary, the selected sphere is moved along a vector pointing towards the central axis of the cylindrical container (Figure 2b). After a series of iterations, the selected sphere reaches its final position, seen represented in 2D (Figure 2d).

**Figure 3:**
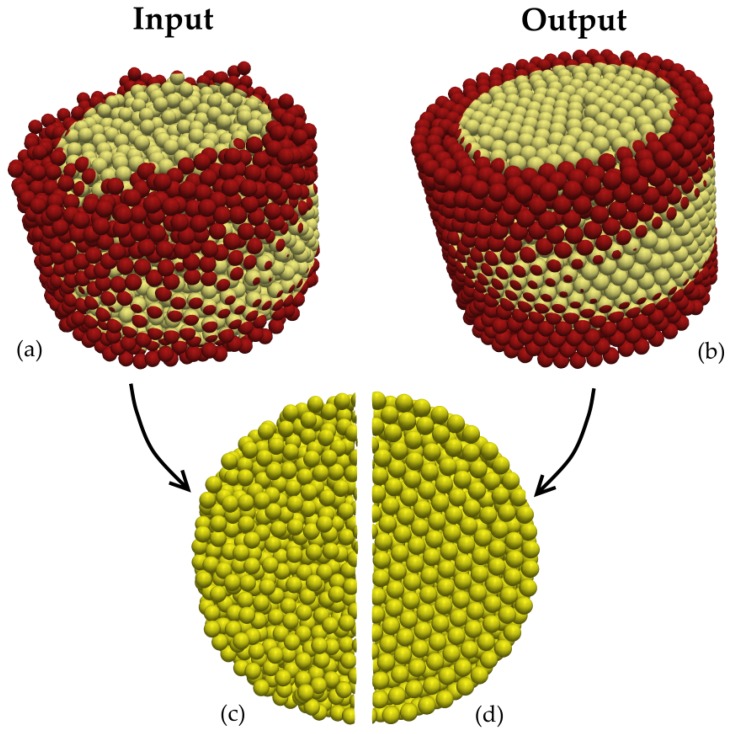
Algorithm input and output example (bi-color applied for visualization enhancement). (**a**) random loose packing input; (**b**) close random packing output; (**c**) mid-height cross-section of very loose packing; (**d**) mid-height cross-section of close random packing.

Figure 3 illustrates a very loose packing (ρ=0.58) input and corresponding close dense random sphere packing (ρ=0.62) output, in 3D and (x−y) plan view (Figure 3c,d). It can be noted that, as the packing approaches maximum density, the structure becomes more orderly with a predominance of face-centered cubic and hexagonal arrangements. While less evident, the slight increase in sphere diameter (4.5%) resulting from the method is also perceptible.

### 2.2. Method Validation

**Figure 4:**
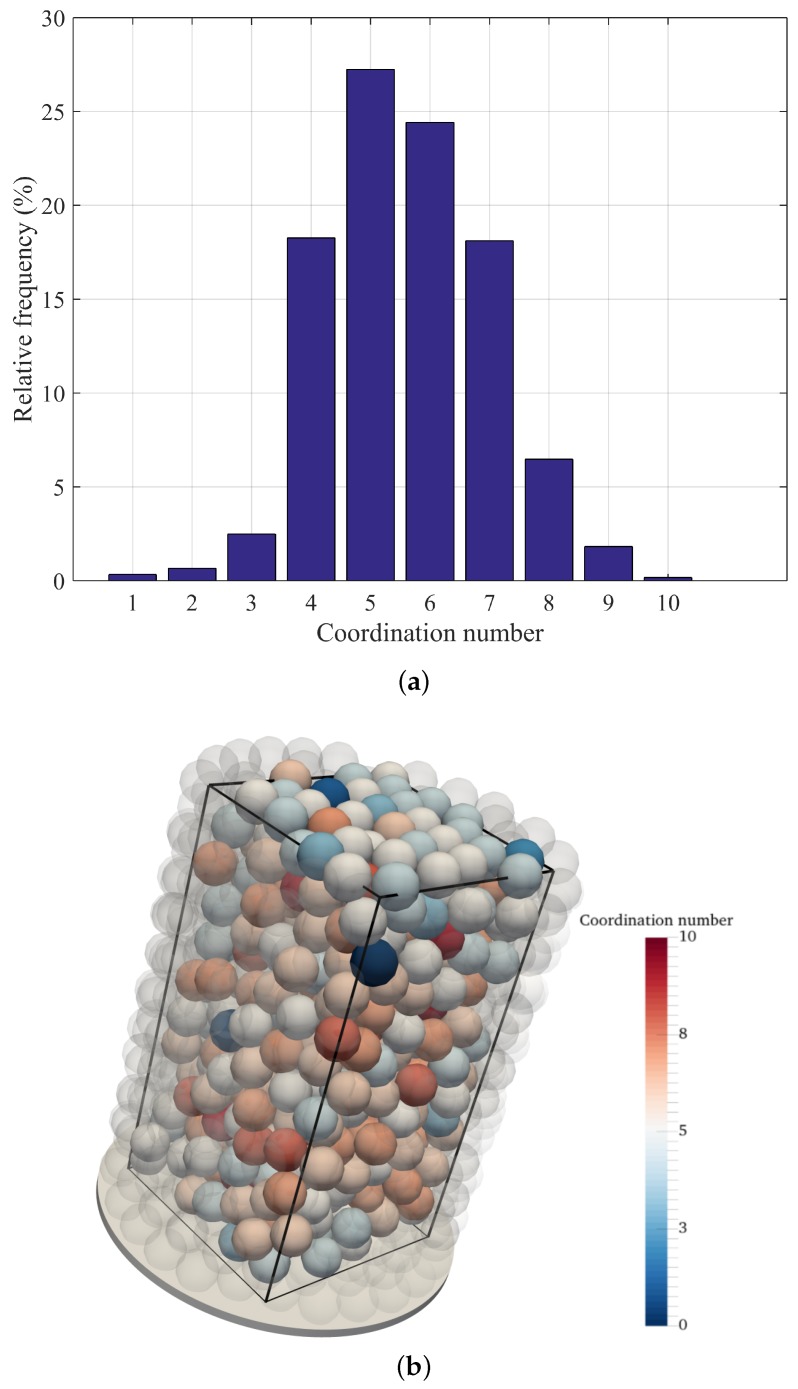

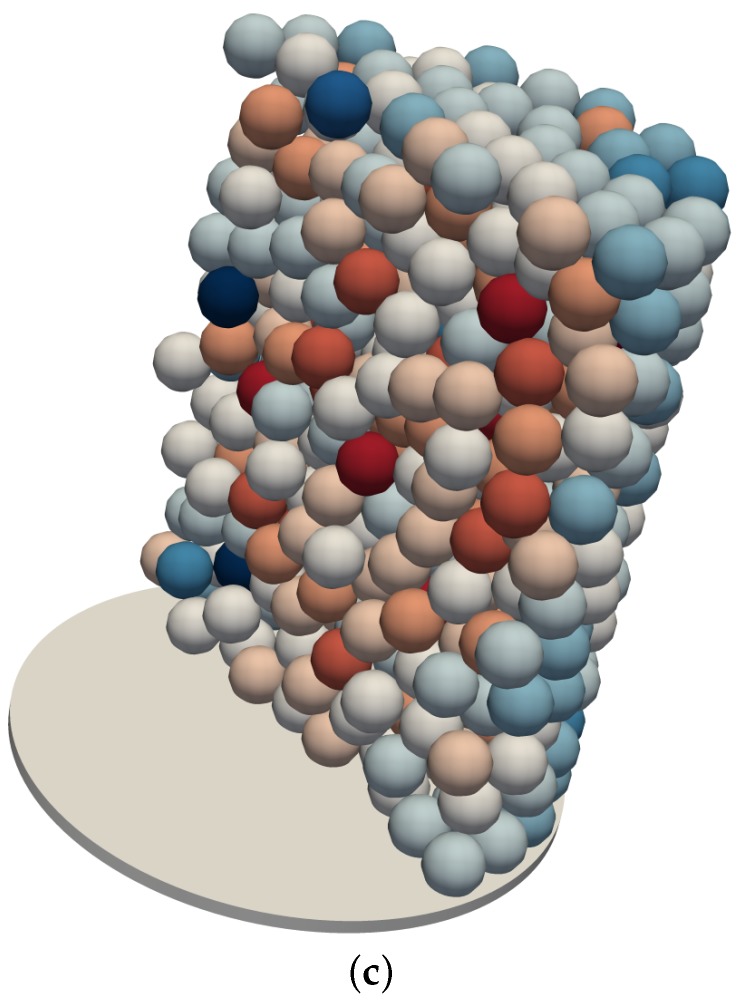
Packing coordination number. (**a**) statistical distribution; (**b**) packing within virtual container with cutoff of two particle diameters from cylindrical boundary; (**c**) diametric section of the same packing.

Statistical mechanics studies [49,50,51] have demonstrated that a packed bed is only physically possible when its solid fraction, as a function of the average coordination number ρ(Z), lies strictly within the range defined by the random loose packing $\rho_{RLP}\left(Z\right)$ and random close packing $\rho_{RCP}$, which can be calculated as follows:

(1) $$\begin{array}{c} \rho_{RLP}\left(Z\right)\approx \frac{Z}{Z+2\sqrt{3}},\\ \rho_{RCP}=\frac{6}{6+2\sqrt{3}}, \end{array}$$

in which *Z* corresponds to the average coordination number. Therefore, a packing produced by the proposed method can be considered valid by computing ρ(Z) via Equation (1) and verifying that $\rho_{RLP}\left(Z\right)\leq \rho \left(Z\right)\leq \rho_{RCP}$. Here, this calculation is exemplified for a packing classified as poured random (Table 1), in which the portion outside the virtual container, subject to boundary effects, was disregarded (Figure 4). The average coordination number and density were 5.61% and 62.64%, which yield 0.618<0.626<0.634, confirming the packing as an equilibrated structure. Spheres in Figure 4c are color coded according to their respective coordination numbers, revealing the increase of this parameter towards the center of the container.

## 3. Pore Size Distribution Method

The starting stage for the PSD method proposed herein consists of the binary solid-void representation of a granular matrix. This is obtained from the synthetic packings by discretizing the domain in a fine mesh of voxels (the volumetric equivalent of pixels). Each voxel has a size of $\frac{radius_{particle}}{15}$, and is assigned zero, if its center is inside a particle (solid), or one if it is located in a void. The pore space is then identified and characterized following a three step approach: conversion to Euclidean distance field; application of a distinct pore locator; and segmentation. The technique is further described in the ensuing sections.

### 3.1. Euclidean Distance Transform (EDT)

**Figure 5:**
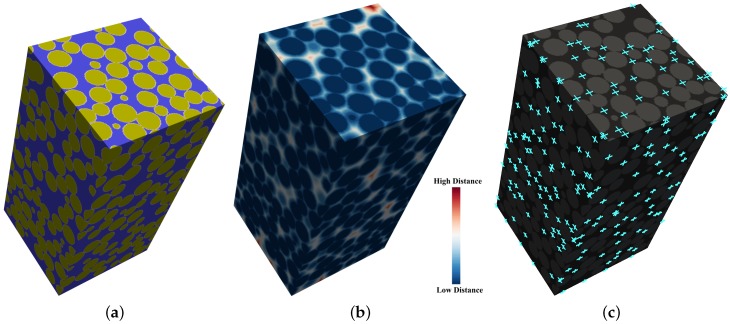
Very loose mono-sized packed spheres. (**a**) binary representation, in which solid and void are represented in yellow and purple, respectively; (**b**) EDT representation; (**c**) local void centers, represented by the light blue crosses.

Distance transforms are image analysis techniques performed on binary representations to map the original data into a more informative field. In the EDT algorithm, solid voxels are considered to be the fixed information and the Euclidean distance from each value is calculated to the nearest fixed value. Figure 5a shows the solid-void binary representation of a virtual container extracted from a very loose random mono-sized packed structure. The specimen was generated in a cylinder with $\frac{radius_{container}}{radius_{sphere}}=10$ and a density value of ρ=57.67%. Figure 5b shows the corresponding EDT map, which serves as input for the next step of the PSD approach.

### 3.2. Local Voids Center

In this step, the EDT representation is subjected to a series of block processing non-maximum suppression operations. The global voxel matrix is scanned with a search cubic block of odd size, in which the central voxel is compared to its neighbors. If its EDT value corresponds to the local maximum, its global index is stored and its neighbors are set to zero to impede recalculation in later stages. The operation is denoted by Equation (2):

(2) $$\arg\max_{x\in S\subseteq X}f\left(x\right):=\left\{x|x\in S\wedge \forall y\in S:f\left(y\right)<f\left(x\right)\right\},$$

where *x* is the void voxel index in the global 3D *X* matrix, *S* is sub-matrix block in *X*, and f(.) is the EDT value. However, moving the search block by its equivalent size does not guarantee stable local maxima, as it is uncertain whether a stored index is a persistent local maximum in a broader context. To this end, the procedure is repeated increasing the search block. For the aforementioned voxel size, only three iterations were necessary with [3×3×3], [5×5×5], and [7×7×7] $\left(\frac{radius_{sphere}}{5},\frac{radius_{sphere}}{3},\frac{radius_{sphere}}{2}\right)$ block sizes. The matrices resulting from each iteration are juxtaposed, and only consistent local maxima are output as true local pore center indices, represented in Figure 5c.

### 3.3. Segmentation

After defining the center of local voids, Equation (3) (minimum Euclidean distance assignment) is applied on given *k* void centers ($c_{1},c_{2},\cdots ,c_{k}$) to segment local pores ($P_{1},P_{2},\cdots ,P_{k}$), which correspond to subsets of void voxel ($x_{v}$).

(3) $$P_{i}=\left\{x_{v}:∥x_{v}-c_{i}{∥}^{2}\leq ∥x_{v}-c_{j}{∥}^{2}\forall j,1\leq j\leq k\right\}.$$

**Figure 6:**
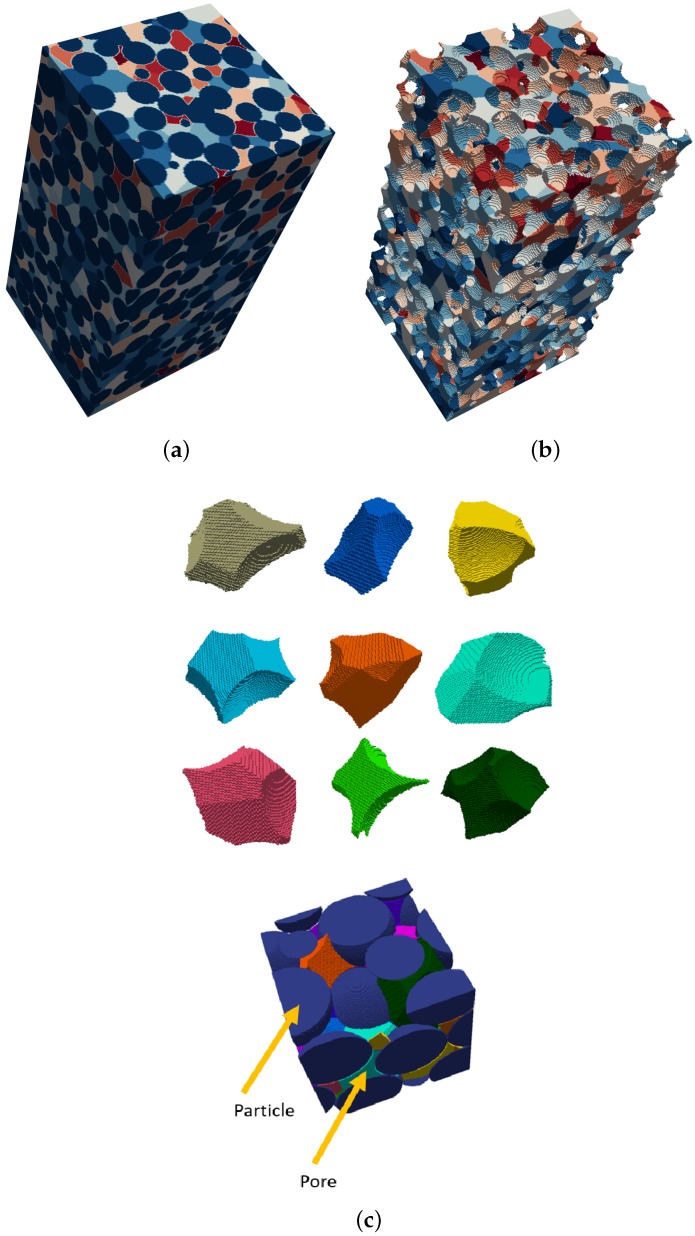
Segmentation result of a very loose mono-sized sphere packing. (**a**) solid and void phases; (**b**) segmented pore space; (**c**) example of extracted local voids.

Figure 6b shows the segmented pore space in a very loosely packed specimen, and Figure 6c shows individual local voids extracted from the sample.

The volume of each individual pore is calculated as the voxel count of that pore multiplied by a voxel volume. For the purpose of the analyses in this paper, the effective pore size is defined as the radius of the sphere of equivalent volume to that of the voxelized pore.

### 3.4. Validation

**Figure 7:**
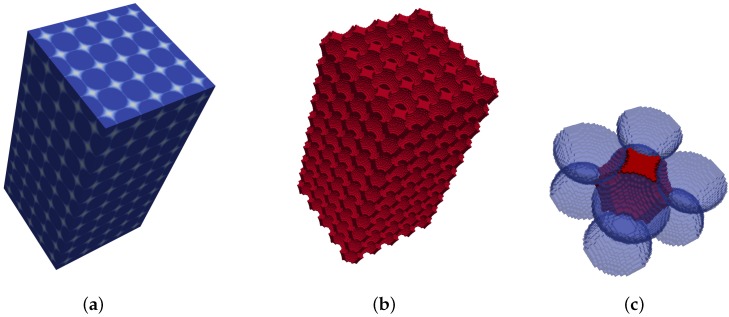
Local void detection in a simple cubic packing. (**a**) EDT representation; (**b**) segmented pore space; (**c**) characteristic local pore.

**Figure 8:**
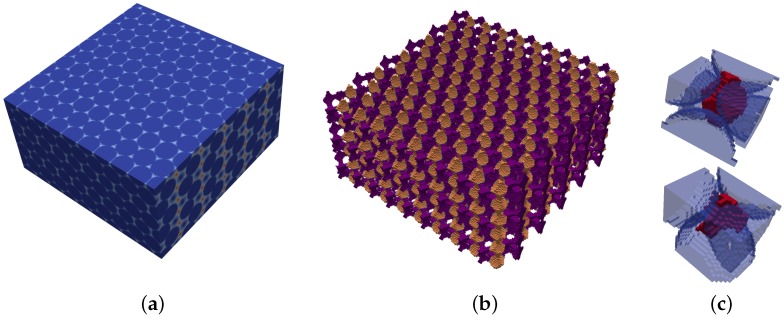
Local void detection in a close hexagonal packing. (**a**) EDT representation; (**b**) segmented pore space; (**c**) characteristic local pores.

The method is validated by applying the proposed approach to a simple cubic packing and to a close hexagonal packing, both of known pore shape and size distribution. The EDT representation and pore space segmentation for the two structures are presented in Figure 7 and Figure 8. Pore segmentation figures are color coded with respect to pore size, providing visual evidence of the method’s precision in partitioning the pore space. Additionally, the precise shape of the voids in these structures is also captured, as illustrated in Figure 7c and Figure 8c. In the simple cubic packing, the effective pore size determined by the proposed method is 0.97r, where *r* is the sphere radius. In a hexagonal packing, there are two major effective pores of 0.43r and 0.58r.

### 3.5. Comparison with Other Methods

In simple cubic packing, a pore is bounded by eight spheres with the effective pore size of 0.97r. With the Delaunay tessellation method, a pore size is defined as the inscribed sphere within a tetrahedron constructed by four spheres, the same pore would be divided in two, each with an effective size of 0.366r, as reported by Gao [13].

**Figure 9:**
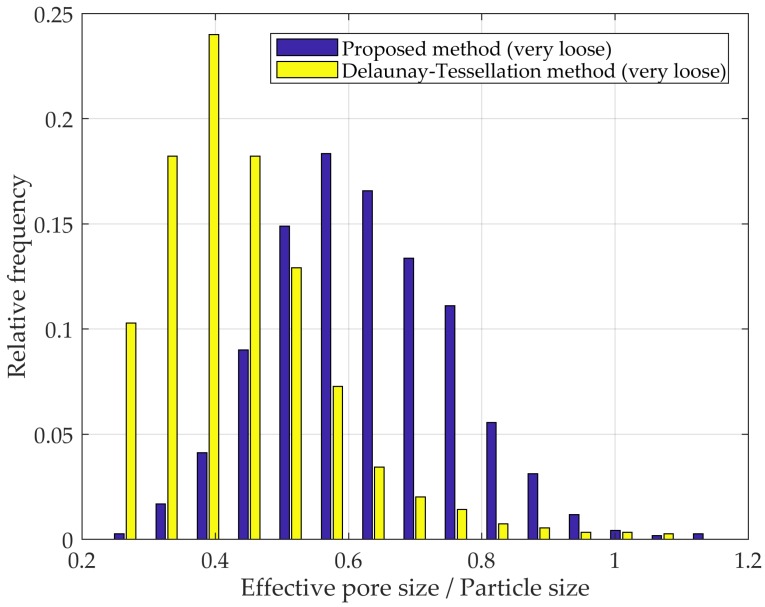
PSD according to the Delaunay tessellation and proposed methods in a very loose mono-sized packed spheres.

Apart from the inherently different pore size definitions—one method backcalculates the effective size from volume, while the other is defined by geometric boundaries—it is still possible to draw insights from comparing the results obtained by the two methods. Figure 9 shows the PSDs of a very loose mono-sized packing, based on Delaunay tessellation [13,19] and the proposed method (EDT-based). In comparing the mean pore size, it can be observed that the EDT-based segmentation yields significantly larger pores, by approximately a factor of 2 compared to Delaunay tessellation results, as expected. However, it is the stark contrast between the shape of distributions, which provides greater insight. In very loose packings, where a broad range of particle arrangements are present, the limited void definition imposed by Delaunay tessellation precludes the method from capturing the full spectrum of pore sizes, as evidenced by the narrow range within which most of the data points are contained.

**Figure 10:**
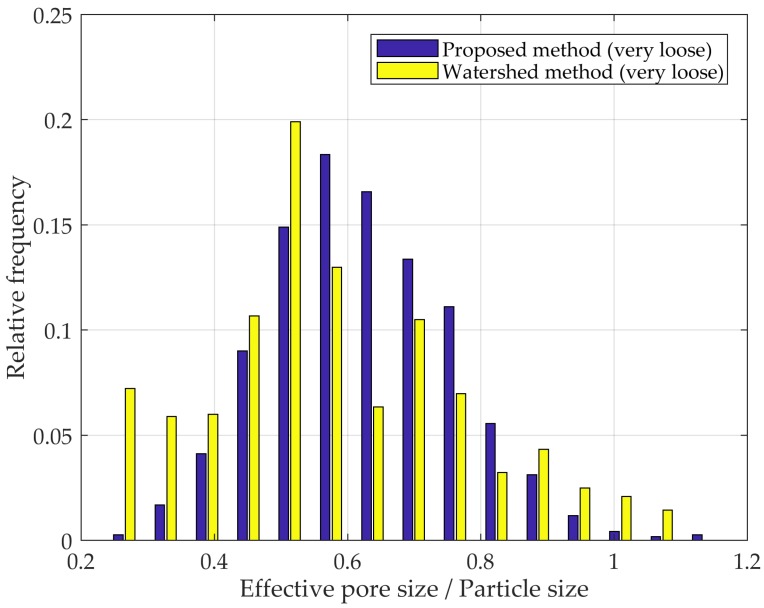
PSD according to the watershed and proposed methods in a very loose mono-sized packed spheres.

The results obtained using the watershed method were more akin to those obtained with the proposed method (Figure 10). Nonetheless, the aforementioned shortcoming of over-segmentation is evidenced by relatively high frequencies of normalized pore sizes smaller than 0.5. Improved results can only be obtained by directly specifying potential catchment basins or thresholding the Euclidian distance field, which were not conducted in this case.

## 4. PSD in Synthetic Microstructure

In this section, the proposed method is applied to an array of packings generated according to Section 2 in cylindrical containers with $\frac{radius_{container}}{radius_{sphere}}=10$. Density states (Table 1), multi-sized packings and boundary effects are evaluated and discussed below.

### 4.1. PSD of Packings at Different Densities

**Figure 11:**
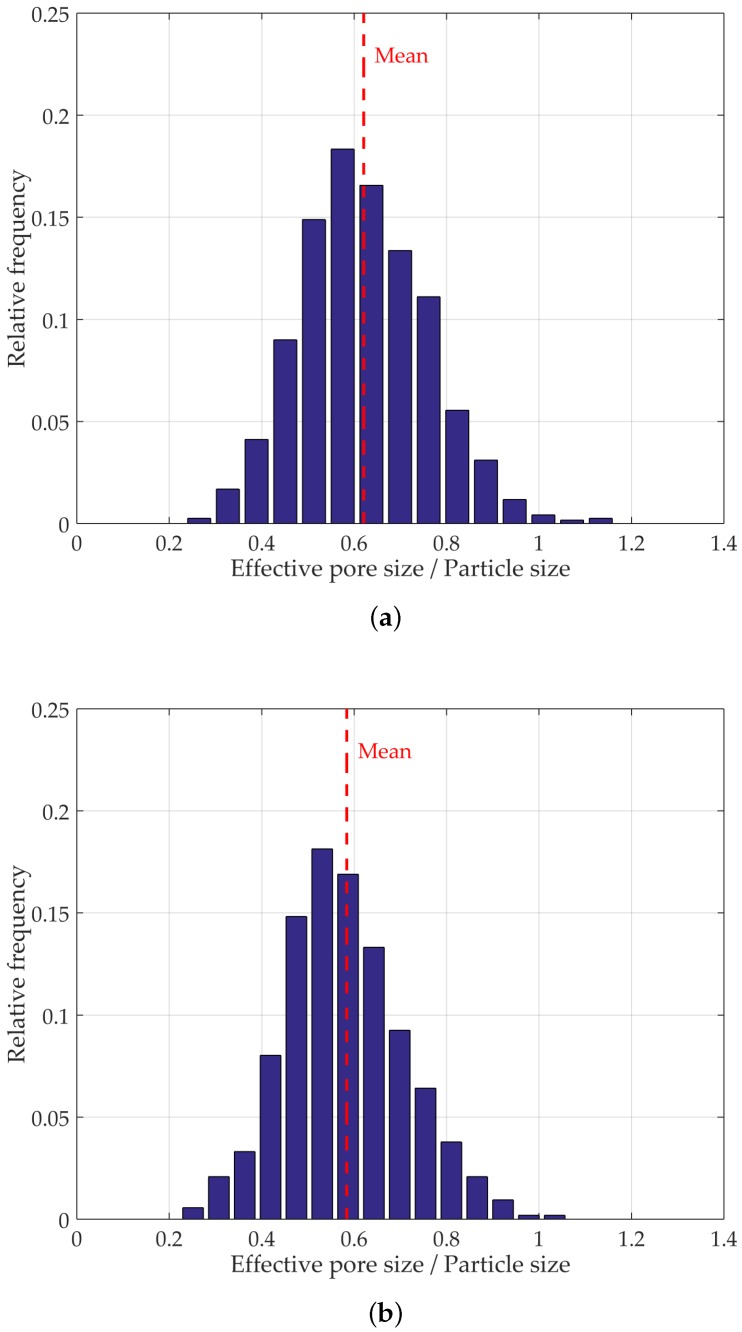

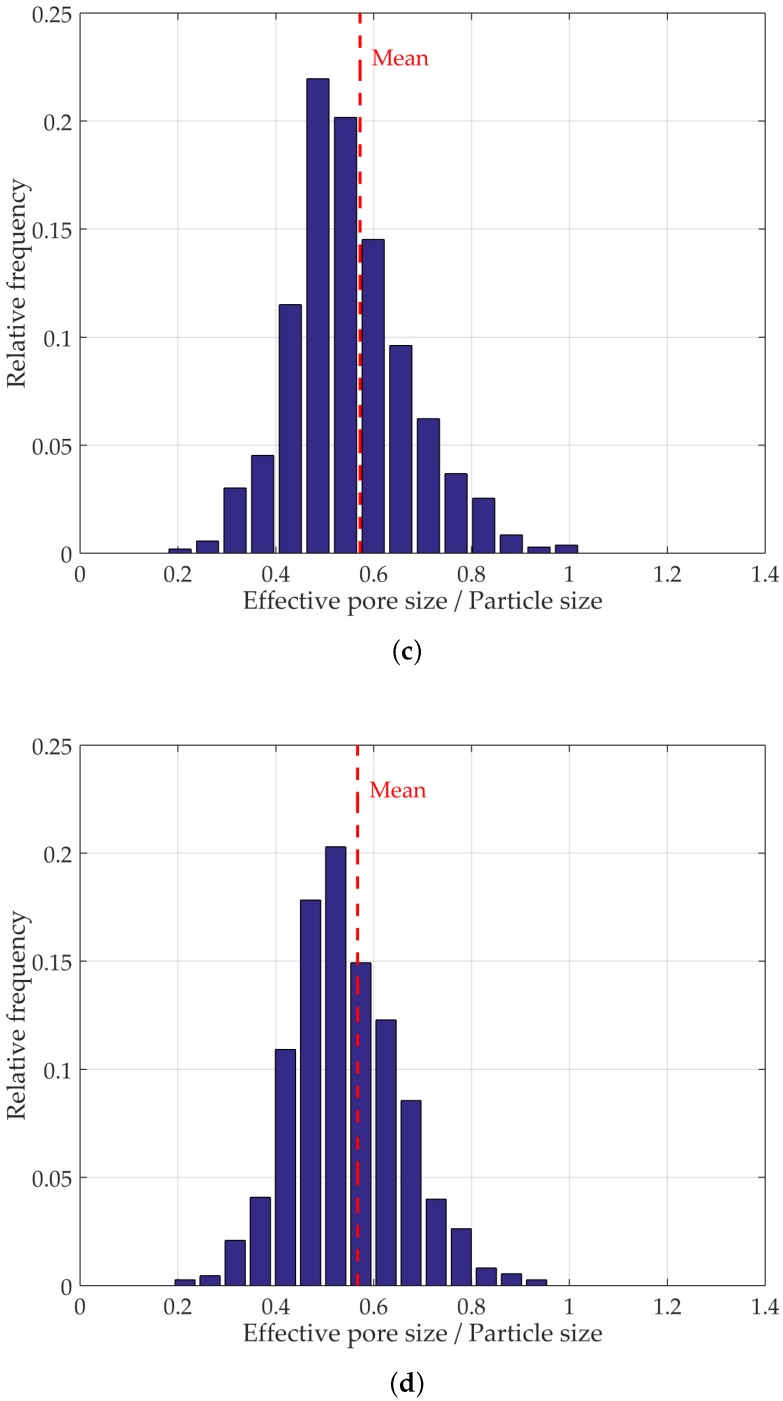
PSD in four different models suggested by Dullien [43]. (**a**) very loose packing; (**b**) loose packing; (**c**) pouring packing; (**d**) dense random packing.

Figure 11 shows the evolution in PSD from very loose random packing to a dense random packing. It can be observed that, as density increases, there is a tendency for the distribution to become more concentrated towards smaller pore sizes, yielding a positively skewed distribution. This is caused both by the overall reduction of the void space, as well as the diminishing number of potential arrangements.

**Figure 12:**
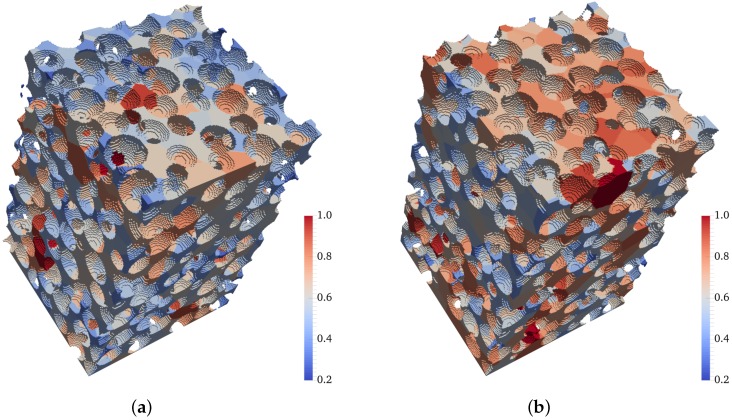
Pore segmentation visualization for four different models (color coded according to normalized pore size). (**a**) dense random packing; (**b**) very loose random packing.

The mean effective normalized pore size for four different granular specimens are 0.567 (dense random packing), 0.572 (poured random packing), 0.584 (loose random packing), and 0.621 (very loose random packing), as presented in Figure 11. These results highlight the effectiveness of the proposed method in characterizing the pore space and revealing the effects of underlying structural changes. Figure 12a,b illustrate the segmented pore space of both dense and very loose packings, respectively, where the overall contrast in pore size is evident.

### 4.2. Pore Shape

**Figure 13:**
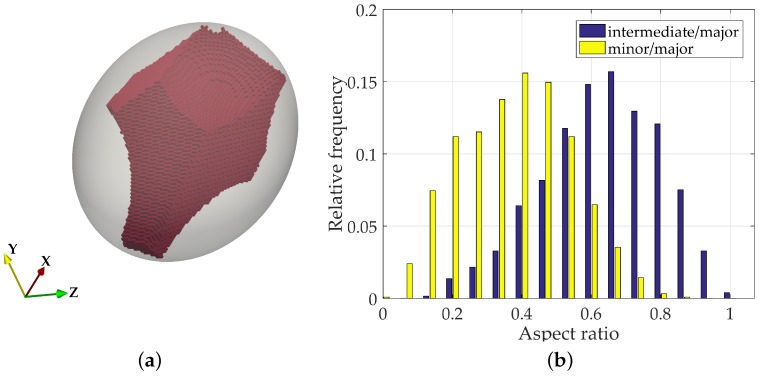
Pore shape distribution. (**a**) example of void and corresponding PCA ellipsoid; (**b**) aspect ratio in dense random packing sample.

No significant differences concerning the distribution of pore shape across different density states was observed. Nonetheless, by calculating the aspect ratio as the quotient between the intermediate and minor axis lengths to the major axis length, it was possible to verify that very few pores present a shape that is close to being spherical. The average aspect ratios of intermediate and minor dimensions to the major length are 0.38 and 0.63, respectively. Therefore, it could be said that on average a void has approximate dimensions 3:2:1 (Figure 13b).

### 4.3. Boundary Effects on PSD Analysis of Multi-Sized Packed Spheres

As discussed previously, the interaction of particles with the boundary results in a looser zone in the immediate vicinity of the container walls. In order to evaluate its effect on the overall PSD, a virtual cylindrical container with a uniform offset of $4\;radius_{particle}$ was extracted from the original container [19]. The analyzed packing consisted of a specimen with particle sizes uniformly distributed in the range of 0.2 to 1, and overall density of ρ=0.58.

**Figure 14:**
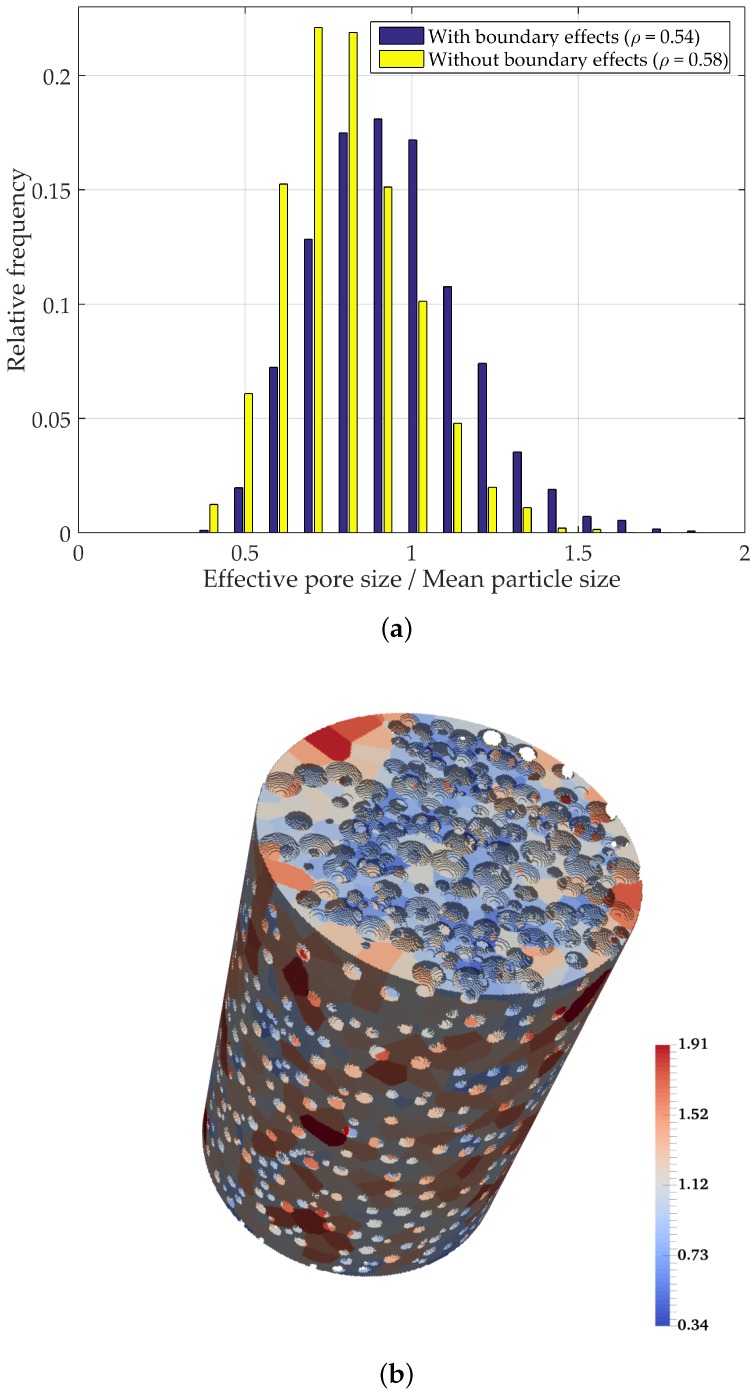

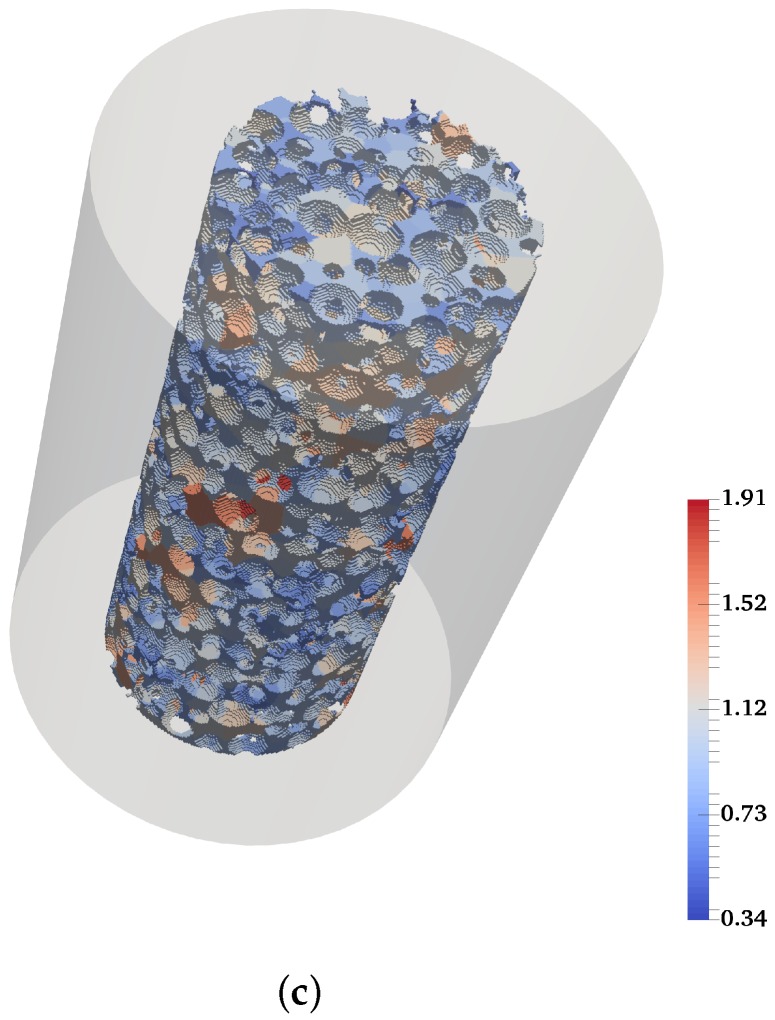
Pore segmentation visualization and PSD for multi-sized packed spheres inside a cylindrical container (color coded according to normalized pore size). (**a**) PSD with and without boundary effects; (**b**) segmented pores with boundary effects; (**c**) segmented pores without boundary affected zone.

By excluding the boundary affected zone, the mean normalized pore size decreased by 10% (from 0.935 to 0.850), as seen in Figure 14. This ratifies the importance of selecting appropriate container sizes, when characterizing a material, so as to minimize the boundary interference on the overall behavior of the material being characterized.

### 4.4. PSD Mono-Sized and Multi-Sized Spheres Packing

**Figure 15:**
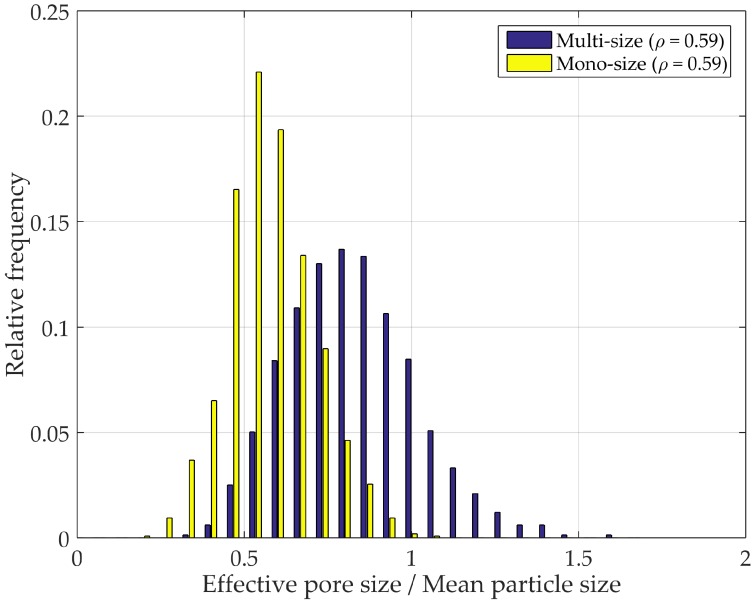
PSD multi mono.

Figure 15 shows PSD in two samples of multi- and mono-sized packed spheres with the same density. The multi-sized sample was generated using the drop-roll approach noted in Section 2.1, with uniform particle size distribution ranging from 0.2 to 1. In order to obtain an equivalent density, a mono-sized sample was generated using the random dense packing method explained in Section 2.1 with a particle radius of 1. Despite the fact that smaller particles tend to fill the voids formed by the larger fraction [19], additional randomness is incorporated in the system by making the packing polydisperse. Different sphere sizes encompass a broader range of potential arrangements, ultimately yielding a broader distribution than that of a mono-sized specimen with the same density (Figure 15).

### 4.5. PSD in Ottawa 20–30 Sand Specimens

**Figure 16:**
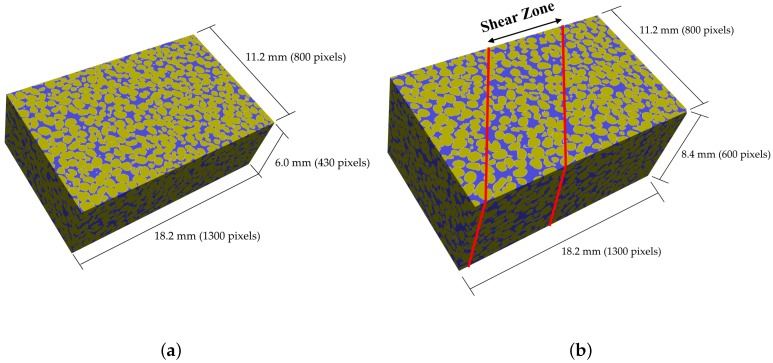
Reconstructed Ottawa 20–30 specimens (**a**) un-sheared and (**b**) sheared [33].

In order to evaluate the performance of the method in an application to real granular materials, 3D digital images of biaxial samples were used as input for the algorithm. These consist of digital reconstructions of sheared and un-sheared specimens of Ottawa 20–30 sand (Figure 16). These were reconstructions obtained by Lu [33], in a procedure involving three major steps: cutting and mounting; grinding and polishing; and imaging and processing.

**Figure 17:**
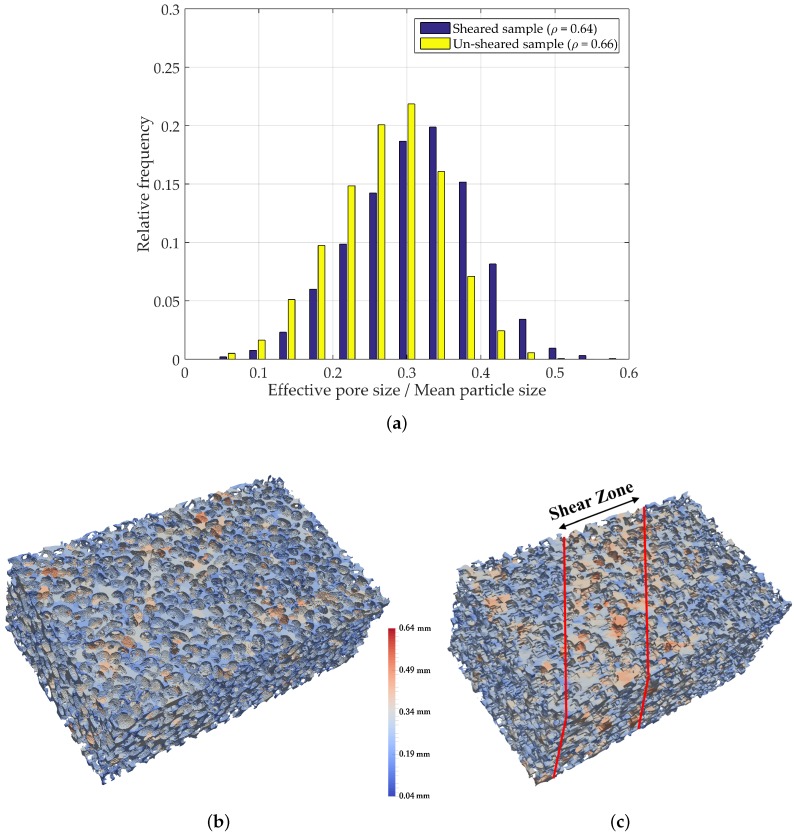
Pore segmentation visualization and PSD for reconstructed un-sheared and sheared Ottawa 20–30 sand. (**a**) PSD for reconstructed un-sheared sample; (**b**) segmented pores for reconstructed un-sheared sample; (**c**) segmented pores for reconstructed sheared sample.

Figure 17a shows PSD for the un-sheared and sheared reconstructed Ottawa 20–30 sand. During the biaxial compression experiment performed by Lu [33] in a dense sample, stress concentrations evolved into a shear band in the specimen. This localization process leads to particles rolling over each other into a looser state (dilation), yielding bigger voids in the shear zone. For the data input, the mean pore size in the entire initial sample increased from 0.2912 mm to 0.3120 mm after shearing. Note that this is based on an average of the entire specimen as opposed to the shear zone only, where the increase would be even more pronounced. The overall PSD undergoes significant changes. Initially constrained between the 0.02 and 0.52 mm range, it expanded considerably in the sheared sample, ultimately lying in the range of 0.04 and 0.64 mm. Figure 17b,c illustrate the segmented pores in the Ottawa sand specimens, color coded based on effective pore size. Note the concentration of larger voids within the shear zone in Figure 17c.

## 5. Conclusions

In this study, a consistent and operator independent technique to determine PSD is proposed that includes the three major steps of EDT domain conversion, non-max suppression, and segmentation. The method has been shown to perform well for binarizations that preserve the key features of the granular matrix. Nonetheless, noisey or poor quality input may affect the success of the algorithm. This limitation could be addressed by pre-processing the input with a variety of operations, such as erosion and dilation. The main features of the proposed approach are:

- No user defined parameters are required;
- It can be applied in any type of microstructure generated synthetically or experimentally;
- Particle shape is not a constraint in this method;
- Pore centers and polyhedral shapes of pores are calculated;
- It can capture the PSD variations for even small changes in a microstructure.

## Figures and Tables

**Table 1:** Density states of packing models, adapted from Dullien [43].

Model	Description	Packing Density
Very loose random packing	Spheres slowly settled	0.56
Loose random packing	Dropped into bed or packed by hand	0.59 to 0.60
Poured random packing	Spheres poured into bed	0.609 to 0.625
Dense random packing	The bed vibrated	0.625 to 0.641
